# The efficiency and safety of zero-exchange workflows in pulsed field ablation: Comprehensive insights from the DISRUPT-AF registry

**DOI:** 10.1016/j.hroo.2025.07.014

**Published:** 2025-07-26

**Authors:** Robert Eckart, Robert Brewer, Devi Nair, Jonathan Dukes, John Costello, Matthew D. Martens, Saja Al-Dujaili, Brad S. Sutton, Jose Osorio, Amin Al-Ahmad, DISRUPT-AF Investigators

**Affiliations:** 1 HCA Florida, Heart Specialists of Sarasota, Sarasota, Florida; 2 MercyOne Iowa Heart Center, West Des Moines, Iowa; 3 Arrythmia Research Group, St. Bernards Healthcare, Jonesboro, Arkansas; 4 Community Memorial Hospital, Ventura, California; 5 Cardiovascular Associates of the Delaware Valley, The Heart House, Haddon Heights, New Jersey; 6 Boston Scientific Corporation, Marlborough, Massachusetts; 7 HCA Florida Hospitals, Mercy Hospital, Miami, Florida; 8 Texas Cardiac Arrhythmia Institute, Austin, Texas

**Keywords:** Atrial fibrillation, Catheter ablation, Transseptal puncture, Pulsed field ablation, Workflow, Zero-Exchange, Zero-Fluoroscopy

## Abstract

**Background:**

Pulsed field ablation (PFA) has revolutionized catheter ablation procedures in terms of safety, efficiency, and efficacy. Streamlined zero-exchange workflows with therapy sheath integrated transseptal puncture (TSP) devices may offer further improvements. However, the impact of device exchanges on real-world PFA procedures remains unknown.

**Objective:**

We aimed to describe and quantify procedural characteristics associated with different PFA workflows.

**Methods:**

The DISRUPT-AF registry (NCT06335082) is an observational, prospective study assessing clinical experience with the pentaspline PFA system in the United States. Procedures are performed as per standard-of-care, where specific workflows and left atrial (LA) access devices were not mandated.

**Results:**

A total of 873 cases (mean age, 68.0 ± 11.4 years; 38.6% women) with 62 unique operators across 20 centers were assessed. Of these, 10.1%, 63.1%, and 26.8% reported “0”-, “1–2”-, and “3+”-exchanges, respectively. Regardless of atrial fibrillation subtype, zero-exchange workflows were associated with significantly shorter and more consistent TSP times (0:5.7 ± 4.2 min; 1–2:15.3 ± 7.3 min; 3+:19.2 ± 9.3 min; *P <*0.001), left atrium dwell times (0:26.9 ± 10.2 min; 1–2:36.8 ± 13.2 min; 3+:50.8 ± 17.8 min; *P <* .001), and relative procedure times (0: 100%; 1–2: 152.7%; 3+: 209.4%; *P <* .001), when compared with 1–2 and 3+-exchange procedures. Despite this increased efficiency, there were no differences in TSP success rates, PFA lesion count, acute pulmonary vein isolation rates, and procedural complication rates between the 3 workflows.

**Conclusion:**

The adoption of zero-exchange workflows enabled by therapy sheath-integrated TSP devices results in significant improvements in procedural efficiency and predictability without compromising patient safety or acute outcomes.

**Key Findings**

▪ In clinical practice, zero-exchange pulsed field ablation workflows facilitated by VersaCross Connect enable more efficient and predictable left atrial access than workflows involving exchanges.
▪ Zero-exchange workflows significantly reduce procedure times, largely owing to the streamlined workflow itself rather than the disease state.
▪ Zero-exchange approach with VersaCross Connect achieves left atrial access effectively, regardless of fluoroscopic guidance.

## Introduction

Catheter ablation has been established as an effective modality for the treatment of atrial fibrillation (AF).^1^^,^^2^ The recent introduction of pulsed field ablation (PFA) has resulted in significant improvements in patient safety, as well as procedural efficacy and efficiency.^3^, ^4^, ^5^, ^6^ Although the adoption of PFA has improved ablation procedures, realizing the full efficiency and safety benefits of PFA is currently limited by the workflows employed in clinical practice.

Inherent to the success of any catheter ablation procedure is safe and effective left atrium (LA) access, achieved by transseptal puncture (TSP), followed by introduction of the therapy sheath and catheter. To achieve this, conventional workflows use a needle and standalone dilator, followed by multiple guidewire and sheath exchanges.^7^^,^^8^ Technological advances to streamline workflows, including purpose-built radiofrequency (RF) wires for TSP, have eliminated guidewire exchange steps but still require a standalone dilator.^8^^,^^9^ Owing to the risk of air embolism or thrombus introduction and procedural inefficiency associated with exchanges, efforts are now focused on eliminating these steps altogether.^10^, ^11^, ^12^ To this end, zero-exchange workflows have recently been enabled by the development of purpose-built options like the VersaCross Connect (VCC) access solution (Boston Scientific), which employs a dedicated RF wire and integrated dilator to facilitate TSP directly through the therapy sheath.

Although these different workflows may allow for an even greater degree of efficiency and effectiveness during PFA procedures, very little is known about their effects on actual clinical practice. Thus, this study aimed to assess the impact of zero-exchange workflows with VCC in real-world PFA procedures.

## Methods

### Study overview and population

The DISRUPT-AF (NCT06335082) is an observational, prospective, multi-center, non-randomized, real-world registry of centers performing PFA after regulatory approval of the pentaspline PFA system (FARAPULSE, Boston Scientific). The study was conducted in accordance with the Declaration of Helsinki. DISRUPT-AF was approved by the appropriate institutional review boards at each site prior to enrollment, and informed consent was obtained from each patient prior to the collection of any data. The data form was developed with the goal of collecting comprehensive information on clinical experience, including procedural efficiency, safety, and long-term effectiveness of PFA. At the time of index procedure, workflow-specific data were obtained, including how many catheter exchanges were required. A catheter exchange was defined as a cycle of a catheter in and out of the LA.

Specific to the study population, DISRUPT-AF is a study enrolling up to 2000 patients with AF at 20 sites across the United States between April 2024 and present. Inclusion criteria include that patients must both be undergoing a de novo ablation procedure and have an indication for AF ablation. Patients currently receiving inotropic or mechanical support were excluded from enrollment. For the final analysis, for patients whom no catheter exchange data were collected on the case report form, and/or where the TSP device could not be determined, were excluded. Additionally, patients in the zero-exchange cohort, where VCC for FARADRIVE was not used, were excluded from the final analysis.

### The pulsed field ablation procedure

PFA procedures were performed in accordance with operator preference and institutional standard-of-care, including sedation technique, procedural guidance, and method for obtaining LA access. TSP devices included NRG (Boston Scientific), BRK (Abbott), VCC for FARADRIVE (Boston Scientific), and VersaCross (Boston Scientific).

The PFA system has been previously described.^4^^,^^13^^,^^14^ Generally, the 12F over-the-wire pentaspline PFA catheter (FARAWAVE; Boston Scientific) is advanced through a 13F steerable sheath (FARADRIVE; Boston Scientific) into the LA. The PFA catheter is positioned at the ostium of each pulmonary vein, and a total of 8 pulsed field lesions are applied per vein (4 in “basket” and 4 in “flower” formation), rotating the catheter between each lesion. For non-pulmonary vein ablation, the catheter was placed into a flower configuration to deliver overlapping sets of pulses at each location. The voltage amplitude ranged between 1.8 and 2.0 kV for lesion creation. All procedures used echocardiography (intracardiac or transesophageal) to assess for the presence of a pericardial effusion post-ablation. Importantly, physicians followed their individual standard-of-care workflows, which may vary.

### Study data specifics

The case report form was composed of questions covering the following areas: baseline patient characteristics, procedural parameters, and complications. TSP time (from femoral access) and TSP success were collected. Success was defined as the TSP device being able to access the LA without multiple puncture attempts or bailout to another device. “Time to FARAWAVE in the LA” is a calculated value where LA dwell time was subtracted from the end-of-procedure time to estimate the time at which the FARAWAVE catheter entered the LA. Major complications were defined as complications causing death, prolonged hospitalization, and/or intervention, and were assessed in terms of the degree of relatedness to the PFA procedure (eg, not related, possibly related, probably related, and definitely related); only complications with a relatedness of “possibly related” or higher were included in the analysis.

### Data analysis

Descriptive statistics in tables present continuous variables as mean ± standard deviation and categorical variables as percentages. Violin plots display data as median ± quartiles, with the width indicating data density. Statistical outliers were identified using the robust regression and outlier removal method with a Q coefficient of 1%. Significance was assessed using one-way analysis of variance (ANOVA) with Holm-Šidák multiple comparison test or student’s t-test, where appropriate, whereas F-tests were used to compare population variances between groups. A *P* < .05 was considered statistically significant. Statistical analysis was performed using GraphPad Prism (version 10.2.3).

## Results

### Study population and overall patient characteristics

**Figure 1:**
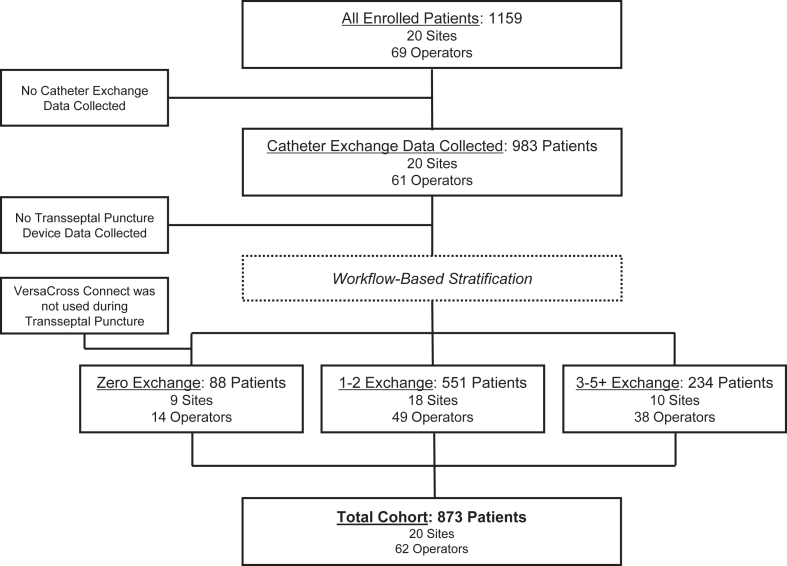
Workflow stratification in the DISRUPT-AF registry. Note: Sites and operators employ different workflows in their individual practices, depending on patient need, and thus can appear in more than 1 workflow cohort.

At the time of this analysis, 1159 patients were enrolled in the DISRUPT-AF registry, and a total of 873 patients were included in this analysis following workflow stratification and exclusions (Figure 1). Procedures were performed at 20 different sites, with 62 unique operators across the United States. The mean patient age was 68.0 ± 11.4 years, of which 38.6% were female (Table 1). The types of AF treated were paroxysmal (57.4%), persistent (38.1%), and long-standing persistent (3.9%). Detailed baseline characteristics and medical history are presented in Table 1.

**Table 1:** Overall patient characteristics

	Full patient cohort
Number of patients	874
Characteristic	
Sex (% female)	38.6
Age (years)	68.0 (±11.4)
BMI (kg/m^2^)	30.5 (±6.7)
Cardiac function (% LVEF)	56.3 (±9.9)
LA diameter (cm)	4.1 (±0.8)
LA volume (mL)	67.8 (±31.9)
Atrial fibrillation	
Paroxysmal, n (%)	501 (57.4)
Persistent, n (%)	333 (38.1)
Long-standing, n (%)	39 (4.5)
Atrial flutter, n (%)	34 (3.9)
AAD use, n (%)	264 (38.8)
OAC use, n (%)	815 (93.2)

Data are represented as mean (±SD) or n (%).

AAD = antiarrhythmic drug; BMI = body mass index; LA = left atrium; LVEF = left ventricular ejection fraction; OAC = oral anticoagulant; SD = standard deviation.

### Patient characteristics following workflow stratification

Following a workflow-based stratification of the patient population, there were 88 patients in the zero-exchange cohort, 551 patients in the 1–2 exchange cohort, and 234 patients in the 3+ exchange cohort (Figure 1). Most parameters assessed were similar between the groups, including age, body mass index, cardiac function (assessed by left ventricular ejection fraction), LA diameter, and LA volume (Table 2). Of note, while the proportions of patients with paroxysmal and persistent AF were the same between the 3 groups, the 3+ exchange cohort had significantly more patients with long-standing AF than the other 2 groups (Table 2).

**Table 2:** Patient characteristics by PFA workflow

	Number of catheter exchanges			*P*-value
	0	1–2	3+	
Number of patients	89	551	234	
Characteristic				
Sex (% female)	38.6	38.0	40.0	ns
Age (years)	68.5 (±11.2)	68.1 (±11.2)	67.5 (±11.8)	ns
BMI (kg/m^2^)	31.2 (±7.4)	30.7 (±6.6)	29.8 (±6.6)	ns
Cardiac function (% LVEF)	58.9 (±7.9)	55.9 (±10.4)	56.2 (±9.3)	ns
LA diameter (cm)	4.0 (±0.8)	4.2 (±0.7)	4.1 (±0.8)	ns
LA volume (mL)	65.7 (±27.2)	66.4 (±35.8)	72.1 (±26.2)	ns
Atrial fibrillation, n (%)				
Paroxysmal	58 (65.9)	320 (58.1)	123 (52.6)	ns
Persistent	28 (31.8)	216 (39.2)	89 (38.0)	ns
Long-standing	2 (2.3)	15 (2.7)	22 (9.4)	<.001^†,‡^
Atrial flutter, n (%)	5 (5.9)	16 (2.9)	13 (5.5)	ns
AAD use, n (%)	26 (44.1)	166 (38.4)	72 (38.1)	ns
OAC use, n (%)	75 (85.2)	511 (92.9)	229 (97.0)	<.001∗^,†,‡^

Data are represented as mean (±SD) or n (%). Ordinary one-way ANOVA with Holm-Sidak multiple comparison test used to compare differences in mean between groups. Statistics performed in GraphPad Prism.

Statistical comparisons: (∗) indicates *P* < .05 between “0” and “1–2”, (†) indicates *P* < .05 between “0” and “3+”, (‡) indicates *P* < .05 between “1–2” and “3+”

AAD = antiarrhythmic drug; ANOVA = analysis of variance; BMI = body mass index; LA = left atrium; LVEF = left ventricular ejection fraction; ns = not significant; OAC = oral anticoagulant; SD = standard deviation.

### Procedural characteristics

**Figure 2:**
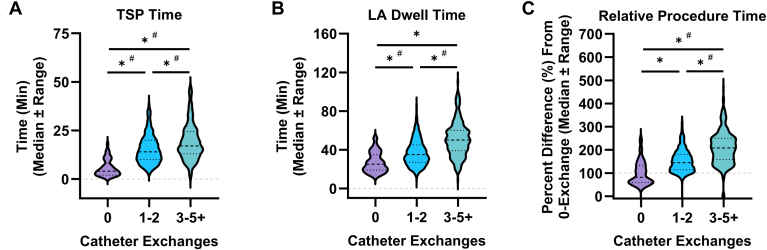
Procedural efficiency and efficacy associated with real-world pulsed field ablation workflows. Shown are transseptal puncture (TSP) time **(A)**, left atrial (LA) dwell time **(B)**, and difference in procedure times relative to zero-exchange workflows **(C)** partitioned by number of catheter exchanges reported during the PFA procedure. *Purple*, characteristics of zero-exchange workflows (indicated as “0;” n = 88); *light blue*, characteristics of 1–2 exchange workflows (indicated as “1–2;” n = 551); *Teal*, characteristics of 3+ exchange workflows (indicated as "3+;” n = 234). Data represented as median ± quartiles. (ns) indicates *P* > 0.05, and (∗) indicates *P <* 0.05 by one-way ANOVA with Holm-Šídák's multiple comparisons test to test differences in mean between groups. (#) indicates *P <* 0.05 by F-Test to compare differences in variability between 2 groups, as indicated.

TSP was successful in all cases, and there were no differences in rates of successful crossing on the first attempt between the 3 workflow groups (Table 3). Zero-exchange workflows were associated with a 62.9% and 70.5% reduction in TSP time, when compared to 1–2 and 3+ exchange workflows, respectively (0: 5.7 ± 4.2 min; 1–2: 15.3 ± 7.3 min; 3+: 19.2 ± 9.3 min) (Figure 2A). Further, zero-exchange workflows were correlated with a 10.6-minute reduction in time between TSP and therapy catheter entering the LA, which was significant when compared to the 3+ exchange group (0: 5.9 ± 4.8 min; 1–2: 8.8 ± 7.9 min; 3+: 16.5 ± 15.2 min) (Table 3). This time savings is reflective of the elimination of the time-consuming TSP apparatus exchanges (eg, needle, sheath, dilator, and guidewire) that are traditionally associated with 3+ exchange workflows.

**Table 3:** Procedural characteristics

	Number of catheter exchanges			*P*-value
	0	1–2	3+	
Number of patients	89	551	234	
Exchanges (mean)	0.0 (±0.0)	1.4 (±0.5)	4.1 (±1.6)	<.001∗^,†,‡^
Transseptal access				
Successful crossing on first attempt (%)	97.8	98.0	97.9	ns
Time to FARAWAVE in LA (from TSP; min, mean)	5.9 (±4.8)	8.8 (±7.9)	16.5 (±15.2)	<.001^†,‡^
Pulsed field ablation				
Total number of lesions (count, mean)	58.9 (±21.4)	62.2 (±18.4)	59.4 (±20.4)	ns
Pulmonary vein lesions	54.5 (±21.5)	45.4 (±15.7)	43.8 (±15.7)	<.001^†,‡^
Non-pulmonary vein lesions	4.4 (±9.3)	16.7 (±16.6)	15.7 (±17.9)	<.001^†,‡^
Acute PVI success (%)	99.7	99.9	100.0	ns
Complications, n (%)	1 (1.1)	6 (1.1)	3 (1.3)	ns
Major complications	1 (1.1)	4 (0.7)	2 (0.8)	ns
Minor complications	0 (0.0)	2 (0.4)	1 (0.4)	ns
Anesthesia				
Anesthesia time (min, mean)	114.9 (±58.5)	100.9 (±26.3)	127.5 (±42.2)	<.001^†,‡^
Imaging				
Fluoroscopy time (min, mean)	8.7 (±6.0)	7.5 (±7.4)	8.6 (±8.5)	ns

Data are represented as mean (±SD) or percent (%). Complications represented as n (%). Ordinary one-way ANOVA with Holm-Sidak multiple comparison test used to compare differences in mean between groups. Statistics performed in GraphPad Prism. Statistical comparisons: (∗) indicates *P <* .05 between “0” and “1–2”, (†) indicates *P <* .05 between “0” and “3+”, (‡) indicates *P <* .05 between “1–2” and “3+”.

ANOVA = analysis of variance; LA = left atrium; PVI = pulmonary vein isolation; SD = standard deviation; TSP = transseptal puncture.

PFA procedures that employed zero-exchange workflows also required significantly shorter time in the LA compared to 1–2 and 3+ exchange procedures (0: 26.9 ± 10.2 min; 1–2: 36.8 ± 13.2 min; 3+: 50.8 ± 17.8 min) (Figure 2B). When overall procedure times relative to zero-exchange workflows were assessed, 1–2 exchange workflows were associated with a 152.7% increase, while 3+ exchange workflows were associated with a 209.4% increase (Figure 2C).

Despite the significant time savings associated with zero-exchange workflows, all 3 groups reported similar numbers of total PFA lesions, acute pulmonary vein isolation (PVI) rates, and procedural complication rates (Table 3, for detailed listing see Supplemental Table 1). Importantly, there were no reports of TSP-related complications, including cardiac tamponade, in any of the 3 workflow groups. Although the study was not powered to assess embolic events, there were no reports of strokes or transient ischemic attacks across the 3 workflow groups (Supplemental Table 1).

Zero-exchange workflows were also associated with significantly more consistent procedure times. This included both TSP time and relative procedure times, where zero-exchange workflows were significantly less variable than 3+ exchange workflows (Figure 2A, Supplemental Table 2).

Our analysis also demonstrated that cases were evenly spread across the 3 workflow groups, with no single site accounting for more than 40.4% of cases in any group (Supplemental Table 3). Of the 20 registry sites, 9 contributed at least 1 zero-exchange case, which involved a total of 14 different operators (Supplemental Table 4).

### Workflow efficiency and consistency in patients with paroxysmal AF

**Figure 3:**
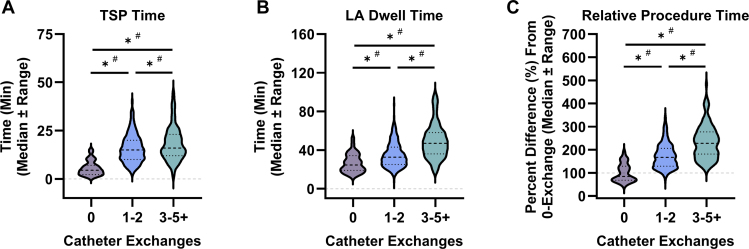
Procedural efficiency and efficacy associated with real-world pulsed field ablation workflows in patients with paroxysmal atrial fibrillation. Shown are transseptal puncture (TSP) time **(A)**, left atrial (LA) dwell time **(B)**, and difference in procedure times relative to zero-exchange workflows **(C)**, partitioned by number of catheter exchanges reported during the PFA procedure in only patients with paroxysmal atrial fibrillation. *Dark purple*, characteristics of zero-exchange workflows (indicated as “0;” n = 57); *Dark blue*, characteristics of 1–2 exchange workflows (indicated as “1–2;” n = 326); *Teal*, characteristics of 3+ exchange workflows (indicated as "3+;” n = 130). Data represented as median ± quartiles. (ns) indicates *P* > 0.05, and (∗) indicates *P <* 0.05 by one-way ANOVA with Holm-Šídák's multiple comparisons test to test differences in mean between groups. (#) indicates *P <* 0.05 by F-test to compare differences in variability between 2 groups, as indicated.

To assess if the differences in workflow observed were due to patient selection, a sub-analysis on the patients with paroxysmal AF was performed. When only considering patients with paroxysmal AF, zero-exchange workflows were associated with shorter TSP times, when compared to 1–2 and 3-+ exchange workflows (0: 5.5 ± 3.7 min; 1–2: 15.9 ± 7.3 min; 3+: 18.3 ± 8.8 min) (Figure 3A). Zero-exchange workflows also required an average of 26.4 ± 9.5 minutes in the LA, which was significantly shorter than 1–2 and 3+ exchange procedures (0: 26.4 ± 9.5 min; 1–2: 34.9 ± 12.6 min; 3+: 49.2 ± 17.7 min) (Figure 3B). Interestingly, controlling for AF subtype, the differences in overall procedure time were more pronounced. To this end, 1–2 exchange and 3+ exchange workflows were associated with a 173.6% and 233.7% increase relative to zero-exchange workflows, respectively (Figure 3C). Remarkably, zero-exchange workflows were also significantly more consistent than both 1–2 and 3+ exchange PFA procedures for every time point assessed in patients with paroxysmal AF (Figure 3A–C).

Despite the increased efficiency observed with zero-exchange PFA procedures in patients with paroxysmal AF, there were no differences in the total number of PFA applications delivered between workflow groups (0: 55.9 ± 18.9; 1–2: 58.6 ± 17.4; 3+: 57.9 ± 21.9). Additionally, no differences in acute PVI success rates between the different workflows were observed (0: 99.6%; 1–2: 99.8%; 3+: 100%).

### Fluoroscopy sub-analysis

No differences were observed in overall fluoroscopy use between the 3 workflows (Table 2). In the zero-exchange group, 75 cases (85.2%) were performed with fluoroscopy, and 13 cases (14.8%) were performed without fluoroscopy.

**Figure 4:**
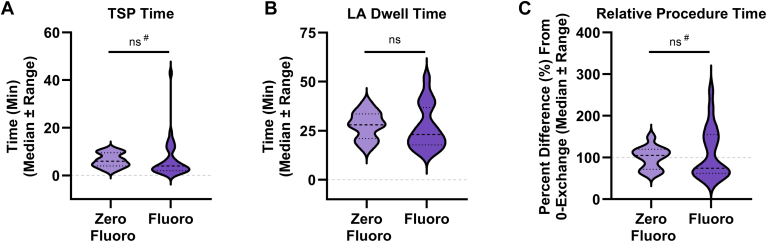
Procedural efficiency and efficacy associated with fluoroscopy-guidance during real-world zero-exchange pulsed field ablation workflows. Shown are transseptal puncture (TSP) time **(A)**, left atrial (LA) dwell time **(B)**, and difference in procedure times relative to zero-fluoroscopy workflows **(C)**, in zero-exchange workflows partitioned by fluoroscopy-guidance reported during the PFA procedure. *Light purple*, characteristics of zero-fluoroscopy workflows (indicated as “Zero Fluoro;” n = 13); *Dark purple*, characteristics of fluoroscopy-guided workflows (indicated as “Fluoro;” n = 75). Data represented as median ± quartiles. (ns) indicates *P* > 0.05 by t-test to compare differences in mean between groups. (#) indicates *P <* 0.05 by F-test to compare differences in variability between groups.

TSP was successful in all cases, and there were no differences in rates of successful crossing on the first attempt between the zero-fluoroscopy and fluoroscopy-guided workflows (Zero Fluoro: 100.0%; Fluoro: 97.3%; *P =* .557). There were also no differences in TSP time, LA dwell time, or relative procedure time between the 2 zero-exchange workflows (Figure 4A–4C). However, we did observe that the time to TSP in zero-fluoroscopy PFA workflows was significantly more consistent when compared to the fluoroscopy-guided workflows (Figure 4A). Although this degree of variability was not carried through to LA dwell time, it significantly impacted the overall procedure time variability (Figure 4B and 4C). This suggests that the imaging modality used during TSP may have an outsized impact on overall procedural consistency.

Despite no differences in LA dwell time or overall procedure time, zero-fluoroscopy workflows were associated with a 25.3% increase in the number of PFA lesions when compared to fluoroscopy-guided workflows (Zero Fluoro: 71.4 ± 19.6; Fluoro: 57.0 ± 21.2; *P =* .030). However, we did not observe any differences in acute PVI success rates between the different workflows (Zero Fluoro: 100.0%; Fluoro: 99.7%; *P =* .676).

## Discussion

PFA has fundamentally altered our collective understanding of what is possible in terms of safety, efficiency, and efficacy for catheter ablation procedures.^3^, ^4^, ^5^ Potential for further improvement in procedural safety and efficiency is thought to be found in streamlined workflows. Despite this, very little is known about the impact of device exchanges in real-world PFA procedures. The DISRUPT-AF registry is an observational, prospective study assessing clinical experience with the pentaspline PFA system, where neither specific workflows nor LA access devices were mandated. The design of this registry uniquely allowed for the analysis of workflows from 873 procedures performed by 62 operators at 20 different sites across the United States. As a result, in this study, we compare the real-world efficiency and efficacy of zero-, 1–2, and 3+ exchange workflows. Additionally, this report provides the first comprehensive description of zero-exchange workflows with therapy sheath-integrated TSP devices.

Central to the success of any PFA procedure is safe and efficient LA access. To this end, we found that zero-exchange workflows with VCC resulted in more efficient and predictable LA access compared to the other workflow groups. We speculate that this efficiency is enabled by therapy sheath-integrated RF wire-based TSP systems, eliminating the need for a starter wire, thus eliminating the need for an exchange step. Additionally, unlike traditional TSP systems, which require transseptal assembly removal and rewiring to reposition on the septum, wire-based systems avoid these steps by tracking the RF wire back up the superior vena cava, followed by the sheath and dilator, and repeating the drop-down. These differences also likely contribute to the greater consistency observed with the zero-exchange workflow than the PFA workflows. Importantly, all workflows resulted in successful LA access, and there were no TSP-related complications reported. To our knowledge, this is the first report to assess the TSP complication rate associated with zero-exchange workflows facilitated by a purpose-built RF wire-based TSP. However, these findings are consistent with a previous study that assessed the use of the VCC system (RF wire and shapeable dilator) during LA appendage occlusion procedures.^15^ These findings also build on the growing body of literature demonstrating the value of purpose-built RF wire-based TSP in terms of safety and efficiency.^8^^,^^16^^,^^17^

We also found that zero-exchange workflows were associated with operators spending less time in the LA. The cumulative impact of zero-exchange workflows was a significant reduction in relative procedure time when compared to 1–2 and 3+ exchange workflows. A sub-analysis of only patients with paroxysmal AF demonstrated that this overall time savings was more associated with workflow than disease state. This is a noteworthy observation, as more advanced AF subtypes are typically associated with longer, more complex procedures that depend heavily on electroanatomic mapping.^18^ This observation also highlights the potential for substantial gains in patient safety and procedural efficiency that may be achieved in the near future with recently introduced “map and ablate” PFA catheters. These technologies could streamline workflows and reduce reliance on complex mapping systems, especially in cases involving advanced AF subtypes. In addition to the efficiency associated with zero-exchange VCC workflows, these procedures were significantly more consistent and predictable compared to 1–2 and 3+ exchange PFA workflows. Although we recognize that the DISRUPT-AF registry was not designed to assess this, it has been suggested that predictability can directly impact patient flow through the healthcare system, as it allows for more reliable scheduling and integration of procedures and ancillary services (ie, anesthesiology, echocardiography, etc.).^19^^,^^20^ This observation builds on the previously described benefits of PFA for health care system efficiency.^5^ Although intriguing, additional studies that directly assess the impact of different PFA workflows on electrophysiology (EP) laboratory throughput are required.

It has previously been suggested that more efficient PFA procedures may also improve both patient and operator safety by reducing ionizing radiation exposure.^5^^,^^21^ To this end, we assessed the impact of more efficient PFA workflows on fluoroscopy use. This analysis revealed that an operator’s choice of workflow had no impact on overall fluoroscopy use. However, it is important to note that the mean fluoroscopy time for the entire study was only 7.9 ± 7.6 minutes, which is significantly less than previous studies and clinical trials involving early adopters of PFA.^3^^,^^5^ This data suggests that with real-world clinical experience, operators have been able to make considerable progress toward fluoroscopy reduction.

Although zero-exchange workflows were not associated with less fluoroscopy use, we did observe that 14.8% of these cases were performed with zero-fluoroscopy. A sub-analysis of these cases demonstrated that zero-exchange workflows can effectively achieve LA access with or without fluoroscopic guidance. Although zero-fluoroscopy workflows with RF-based TSP wires have previously been shown to be feasible, to our knowledge, this is the first demonstration of this using zero-exchange PFA workflows.^22^^,^^23^ However, it is important to note that DISRUPT-AF is a United States-based registry, where zero-fluoroscopy workflows are enabled by easier access to echocardiography-based imaging modalities. Previous reports suggest that fluoroscopy-only workflows, like those common to European centers, may encounter more difficulties, and thus complications, when compared to procedures aided by transesophageal echocardiography.^24^ However, the technologies that enable zero-exchange workflows have only become available recently in these other geographies; thus, further studies will be required in the future.

This sub-analysis of the DISRUPT-AF registry is limited. First, because it is a prospective, observational study, and therefore, patients were not randomized to a particular workflow. As a result, the workflow cohorts are unevenly powered. Second, real-world data sources are limited in that no explicit inclusion and exclusion criteria are used, procedures were performed based on individual centers’ standard-of-care and operator preference, and data are reported by the operator or site; thus, the data can be variable and/or incomplete. Third, while high-level workflow data was collected, comments beyond the number of exchanges, including specific workflow steps and order of steps, cannot be made. Fourth, this study only assesses acute procedural outcomes and complications; further studies are needed to confirm these results and evaluate the long-term outcomes associated with each workflow. Finally, this study specifically examines zero-exchange workflows facilitated by VCC for FARADRIVE. Owing to potential differences between the purpose-built VCC access solutions (ie, dedicated wire, shapeable therapy-sheath integrated dilator, and generator-specific system) and other methods devised to reduce device exchanges, the safety, efficiency, and efficacy observed in this study should not be assumed for other potential zero-exchange workflows.

## Conclusion

This study from the DISRUPT-AF registry provides valuable insights into the efficiency and efficacy of real-world PFA workflows, including the benefits of zero-exchange workflows facilitated by VCC for FARADRIVE. The adoption of zero-exchange workflows resulted in significant improvements in procedural efficiency and predictability without compromising patient safety or acute outcomes. Future studies are required to further explore the impact of different PFA workflows on EP laboratory throughput and long-term procedural success.

## Disclosures

A. Al-Ahmad consults for Boston Scientific. M. Martens, S. Al-Dujaili, and B. Sutton are employees of Boston Scientific and were involved in study conceptualization, data analysis, and manuscript preparation. J. Osorio is President of the Board of Heart Rhythm Clinical and Research Solutions,which receives funding from Boston Scientific to facilitate the DISRUPT-AF registry. The remaining authors have no conflicts to disclose.
