# Supplementary material for: The efficiency and safety of zero-exchange workflows in pulsed field ablation: Comprehensive insights from the DISRUPT-AF registry
Source: Heart Rhythm O2. 2025 Jul 26;6(10):1508–15. doi: 10.1016/j.hroo.2025.07.014 (PMC12570166; doi:10.1016/j.hroo.2025.07.014)
Supplement: Supplementary Tables 1-4 [file mmc1.docx]

**12. DATA SUPPLEMENT**

| **Supplemental Table 1. Procedural Complications** | | | | | | | | | |  |
| --- | --- | --- | --- | --- | --- | --- | --- | --- | --- | --- |
|  |  |  |  | **Number of Catheter Exchanges** | | | | |  | |
|  |  | **Full Cohort** |  | **0** |  | **1-2** |  | **3+** |  | |
| Number of Patients |  | 874 |  | 89 |  | 551 |  | 234 |  | |
| **Major Complications** |  | **7 (0.80)** |  | **1 (1.1)** |  | **4 (0.73)** |  | **2 (0.85)** |  | |
| Myocardial Infarction |  | 1 (0.11) |  | 0 (0) |  | 1 (0.18) |  | 0 (0) |  | |
| Stroke |  | 0 (0.0) |  | 0 (0.0) |  | 0 (0.0) |  | 0 (0.0) |  | |
| Transient Ischemic Attack (TIA) |  | 0 (0.0) |  | 0 (0.0) |  | 0 (0.0) |  | 0 (0.0) |  | |
| Coronary Spasm |  | 1 (0.11) |  | 0 (0) |  | 1 (0.18) |  | 0 (0) |  | |
| Pericarditis |  | 1 (0.11) |  | 0 (0) |  | 1 (0.18) |  | 0 (0) |  | |
| Post-Operative Arterial Injury |  | 1 (0.11) |  | 0 (0) |  | 0 (0) |  | 1 (0.42) |  | |
| Vascular Access |  |  |  |  |  |  |  |  |  | |
| *Hematoma* |  | 1 (0.11) |  | 0 (0) |  | 1 (0.18) |  | 0 (0) |  | |
| *Pseudoaneurysm* |  | 2 (0.23) |  | 1 (1.1) |  | 0 (0) |  | 1 (0.42) |  | |
| **Minor Complications** |  | **3 (0.34)** |  | **0 (0)** |  | **2 (0.36)** |  | **1 (0.43)** |  | |
| Urinary Retention |  | 1 (0.11) |  | 0 (0) |  | 1 (0.18) |  | 0 (0) |  | |
| Elevated Bilirubin |  | 1 (0.11) |  | 0 (0) |  | 1 (0.18) |  | 0 (0) |  | |
| Allergic Reaction at Access Site |  | 1 (0.11) |  | 0 (0) |  | 0 (0) |  | 1 (0.43) |  | |
| *Data are represented as n (%).* | | | | | | | | | |  |

| **Supplemental Table 2. Variability and Procedural Characteristics** | | | | | | | | | | | | | |
| --- | --- | --- | --- | --- | --- | --- | --- | --- | --- | --- | --- | --- | --- |
|  |  | **Number of Catheter Exchanges** | | | | |  | **F- Test p-Value** | | | | |  |
|  |  | **0** |  | **1-2** |  | **3+** |  | **A : B** |  | **A : C** |  | **B : C** |  |
| Number of Patients |  | 89 |  | 551 |  | 234 |  |  |  |  |  |  |  |
| **Time Points** |  |  |  |  |  |  |  |  |  |  |  |  |  |
| TSP Time (from femoral access; min, Mean) |  | 5.7(±4.2) |  | 15.3(±7.3) |  | 19.2(±9.3) |  | <0.001 |  | <0.001 |  | <0.001 |  |
| Time to FARAWAVE in LA (from TSP; min, Mean) |  | 5.9(±4.8) |  | 8.8(±7.9) |  | 16.5(±15.2) |  | <0.001 |  | <0.001 |  | <0.001 |  |
| LA Dwell Time (min, Mean) |  | 26.9(±10.2) |  | 36.8(±13.2) |  | 50.8(±17.8) |  | 0.026 |  | 0.397 |  | <0.001 |  |
| Procedure Time Difference (Relative to 0-Exchange, Mean) |  | 0.0(±21.5) |  | 22.3(±20) |  | 47.2(±28.5) |  | 0.439 |  | 0.018 |  | <0.001 |  |
| Fluoroscopy Time (min, Mean) |  | 8.7(±6.0) |  | 7.5(±7.4) |  | 8.6(±8.5) |  | 0.020 |  | <0.001 |  | 0.014 |  |
| *Data are represented as Mean (± SD). Procedure time is presented as percent (%) difference from zero-exchange.* F-tests used to compare population variances between groups. Statistics performed in GraphPad Prism. Statistical comparisons: (A) indicates “0 Exchange Cohort”, (B) indicates “1-2 Exchange Cohort”, (C) indicates “3+ Exchange Cohort”. | | | | | | | | | | | | | |
| *min, Minute; TSP, Transseptal Puncture; LA, Left Atrium; PVI, Pulmonary Vein Isolation.* | | | | | | | | | | | | | |

| **Supplemental Table 3. Distribution of Workflows Across Sites** | | | | | | | |
| --- | --- | --- | --- | --- | --- | --- | --- |
|  |  | **Number of Catheter Exchanges** | | | | |  |
|  |  | **0** |  | **1-2** |  | **3+** |  |
| Arrhythmia Institute at Grandview |  | 0 (0.0) |  | 60 (10.8) |  | 10 (4.2) |  |
| Arrhythmia Research Group |  | 9 (10.1) |  | 0 (0.0) |  | 0 (0.0) |  |
| Community Memorial Hospital |  | 7 (7.9) |  | 9 (1.6) |  | 7 (2.9) |  |
| Arrhythmia Center of South Florida |  | 3 (3.4) |  | 31 (5.6) |  | 3 (1.3) |  |
| Ascension St. Vincent's Jacksonville |  | 0 (0.0) |  | 18 (3.2) |  | 0 (0.0) |  |
| HCA Florida Mercy Hospital |  | 0 (0.0) |  | 8 (1.4) |  | 0 (0.0) |  |
| Sarasota Memorial Health System |  | 28 (31.5) |  | 1 (0.2) |  | 0 (0.0) |  |
| Endeavor Health (Northshore) |  | 0 (0.0) |  | 5 (0.9) |  | 0 (0.0) |  |
| Mercy One Iowa Heart Center |  | 36 (40.4) |  | 29 (5.2) |  | 4 (1.7) |  |
| University of Iowa |  | 3 (3.4) |  | 31 (5.6) |  | 24 (10.0) |  |
| Brigham and Women’s Hospital |  | 0 (0.0) |  | 2 (0.4) |  | 53 (22.2) |  |
| Cardiovascular Associates of the Delaware Valley |  | 0 (0.0) |  | 75 (13.5) |  | 9 (3.8) |  |
| Mission Hospital |  | 0 (0.0) |  | 28 (5.1) |  | 0 (0.0) |  |
| The Christ Hospital |  | 0 (0.0) |  | 4 (0.7) |  | 0 (0.0) |  |
| OhioHealth Research Institute |  | 0 (0.0) |  | 3 (0.5) |  | 0 (0.0) |  |
| Allegheny Health Network |  | 0 (0.0) |  | 0 (0.0) |  | 49 (20.5) |  |
| HCA Trident Medical Center |  | 1 (1.1) |  | 35 (6.3) |  | 0 (0.0) |  |
| Texas Cardiac Arrhythmia Research Foundation |  | 1 (1.1) |  | 175 (31.6) |  | 62 (29.5) |  |
| St. Mark's Hospital |  | 0 (0.0) |  | 25 (4.5) |  | 0 (0.0) |  |
| HCA Chippenham Hospital |  | 1 (1.1) |  | 15 (2.7) |  | 18 (7.5) |  |
| *Data represented as n (%).* | | | | | | | |

| **Supplemental Table 4. Distribution of Workflows Across Operators at Study Sites** | | | | | | | | | |
| --- | --- | --- | --- | --- | --- | --- | --- | --- | --- |
|  |  |  |  | **Number of Catheter Exchanges** | | | | |  |
|  |  | **Total Operators** |  | **0** |  | **1-2** |  | **3+** |  |
| Arrhythmia Institute at Grandview |  | 2 |  | 0 (0.0) |  | 2 (100.0) |  | 2 (100.0) |  |
| Arrhythmia Research Group |  | 1 |  | 1 (100.0) |  | 0 (0.0) |  | 0 (0.0) |  |
| Community Memorial Hospital |  | 1 |  | 1 (100.0) |  | 1 (100.0) |  | 1 (100.0) |  |
| Arrhythmia Center of South Florida |  | 3 |  | 1 (33.3) |  | 3 (100.0) |  | 3 (100.0) |  |
| Ascension St. Vincent's Jacksonville |  | 2 |  | 0 (0.0) |  | 2 (100.0) |  | 0 (0.0) |  |
| HCA Florida Mercy Hospital |  | 1 |  | 0 (0.0) |  | 1 (100.0) |  | 0 (0.0) |  |
| Sarasota Memorial Health System |  | 1 |  | 1 (100.0) |  | 1 (100.0) |  | 0 (0.0) |  |
| Endeavor Health (Northshore) |  | 1 |  | 0 (0.0) |  | 1 (100.0) |  | 0 (0.0) |  |
| Mercy One Iowa Heart Center |  | 7 |  | 6 (85.7) |  | 6 (85.7) |  | 2 (28.6) |  |
| University of Iowa |  | 5 |  | 1 (20.0) |  | 4 (80.0) |  | 4 (80.0) |  |
| Brigham and Women’s Hospital |  | 5 |  | 0 (0.0) |  | 1 (20.0) |  | 5 (100.0) |  |
| Cardiovascular Associates of the Delaware Valley |  | 2 |  | 0 (0.0) |  | 2 (100.0) |  | 2 (100.0) |  |
| Mission Hospital |  | 2 |  | 0 (0.0) |  | 2 (100.0) |  | 0 (0.0) |  |
| The Christ Hospital |  | 1 |  | 0 (0.0) |  | 1 (100.0) |  | 0 (0.0) |  |
| OhioHealth Research Institute |  | 2 |  | 0 (0.0) |  | 2 (100.0) |  | 0 (0.0) |  |
| Allegheny Health Network |  | 5 |  | 0 (0.0) |  | 0 (0.0) |  | 5 (100.0) |  |
| HCA Trident Medical Center |  | 2 |  | 1 (50.0) |  | 2 (100.0) |  | 0 (0.0) |  |
| Texas Cardiac Arrhythmia Research Foundation |  | 12 |  | 1 (8.3) |  | 12 (100.0) |  | 11 (91.7) |  |
| St. Mark's Hospital |  | 2 |  | 0 (0.0) |  | 2 (100.0) |  | 0 (0.0) |  |
| HCA Chippenham Hospital |  | 4 |  | 1 (25.0) |  | 3 (75.0) |  | 3 (75.0) |  |
| *Data are represented as n (%), indicating the number and percentage of operators at each site employing each workflow type.* | | | | | | | | | |
